# A Real-Time Circuit Phase Delay Correction System for MEMS Vibratory Gyroscopes

**DOI:** 10.3390/mi12050506

**Published:** 2021-04-30

**Authors:** Pengfei Xu, Zhenyu Wei, Zhiyu Guo, Lu Jia, Guowei Han, Chaowei Si, Jin Ning, Fuhua Yang

**Affiliations:** 1 Engineering Research Center for Semiconductor Integrated Technology, Institute of Semiconductors, Chinese Academy of Sciences, Beijing 100083, China; xupengfei@semi.ac.cn (P.X.); zywei97@semi.ac.cn (Z.W.); gzy@semi.ac.cn (Z.G.); jialu@semi.ac.cn (L.J.); ningjin@semi.ac.cn (J.N.); 2 College of Materials Science and Opto-Electronic Technology, University of Chinese Academy of Sciences, Beijing 100049, China; 3 School of Electronic, Electrical and Communication Engineering, University of Chinese Academy of Sciences, Beijing 100049, China; 4 State Key Laboratory of Transducer Technology, Chinese Academy of Sciences, Beijing 100083, China

**Keywords:** MEMS gyroscopes, circuit phase delay, IQ coupling, real-time correction system

## Abstract

With the development of the designing and manufacturing level for micro-electromechanical system (MEMS) gyroscopes, the control circuit system has become a key point to determine their internal performance. Nevertheless, the phase delay of electronic components may result in some serious hazards. This study described a real-time circuit phase delay correction system for MEMS vibratory gyroscopes. A detailed theoretical analysis was provided to clarify the influence of circuit phase delay on the in-phase and quadrature (IQ) coupling characteristics and the zero-rate output (ZRO) utilizing a force-to-rebalance (FTR) closed-loop detection and quadrature correction system. By deducing the relationship between the amplitude-frequency, the phase-frequency of the MEMS gyroscope, and the phase relationship of the whole control loop, a real-time correction system was proposed to automatically adjust the phase reference value of the phase-locked loop (PLL) and thus compensate for the real-time circuit phase delay. The experimental results showed that the correction system can accurately measure and compensate the circuit phase delay in real time. Furthermore, the unwanted IQ coupling can be eliminated and the ZRO was decreased by 755% to 0.095°/s. This correction system realized a small angle random walk of 0.978°/√h and a low bias instability of 9.458°/h together with a scale factor nonlinearity of 255 ppm at room temperature. The thermal drift of the ZRO was reduced to 0.0034°/s/°C at a temperature range from −20 to 70 °C.

## 1. Introduction

Micro-electromechanical system (MEMS) gyroscopes, measuring the angular rate motion based on the Coriolis effect [1], have been widely adopted for industrial and consumer applications [2,3] for their small size, high integration, low cost, and low power consumption. With the rapid development of electronic techniques, the performance of MEMS gyroscopes has been considerably improved, bringing a new future for potential military applications [4,5,6,7].

Vibratory gyroscopes work through Coriolis coupling between two orthogonal modes where one mode is forced to oscillate along the drive axis while the other operates as an accelerometer sensing the angular rate-induced Coriolis acceleration along the sense axis [8,9]. Limited by manufacturing imperfections and immature packaging techniques, in-phase and quadrature error, caused by damping and stiffness coupling, are inevitable for vibratory gyroscopes [10]. Theoretically, the desired angular rate and unwanted quadrature error signals are orthogonal and could be separated via a phase-sensitive demodulation method because they are respectively modulated by the drive velocity and displacement [11]. However, the gyroscope cannot work in the resonant state when circuit phase delay (generated by ADC, DAC, C/V, etc.) exists [12]. In this case, the quadrature error is not −90° out of phase with the angular rate signal, introducing a skew between the quadrature and in-phase channel and hence causing in-phase and quadrature (IQ) coupling [13,14].

The existence of quadrature errors seriously affects the performance of gyroscopes, especially the dynamic range, bias stability, and temperature sensitivity [15,16]. To eliminate the unwanted quadrature error signals, the quadrature cancellation technique is widely used in the closed-loop control electronics to improve bias instability (BI), the angle random walk (ARW), the linearity of the scale factor, and the dynamic range of the interface circuit [17,18,19]. However, this technique is sensitive to phase error for these two channels. Besides, its effectiveness is greatly limited by the stability and accuracy of the phase relationship between the demodulation and the sense signal [20,21]. It was proved that open-loop detection and different phase compensation methods can effectively compensate the errors caused by circuit phase delay, but they are inapplicable to most gyroscope control circuits [11,13]. A one-time off-line frequency sweep procedure was proposed to eliminate the in-phase Coriolis and the quadrature signal [22], and the circuit phase delay was compensated by modifying the reference value of the phase-locked loop (PLL). Nevertheless, this method cannot always accurately reflect the true circuit phase delay. A novel procedure, force-to-rebalance (FTR) control, has been used to calculate the total phase delay within the working process by adopting a digital all-pass filter to realize a one-time phase self-compensation [1]. No real-time phase delay correction method has been mentioned until now, and there is a need for further research to realize real-time circuit phase delay correction in order to improve the performance of MEMS gyroscopes.

In this work, we demonstrated a real-time circuit phase delay correction system to compensate the unwanted circuit phase delay for MEMS vibratory gyroscopes. The influence of circuit phase delay on the IQ coupling characteristics and the zero-rate output (ZRO) was analyzed via an FTR closed-loop detection and quadrature correction system. Meanwhile, a detailed theoretical analysis for the effect of circuit phase delay on the in-phase and quadrature channels was given. Based on the amplitude-frequency and the phase-frequency characteristics of MEMS gyroscopes and the phase relationship of the whole control loop, we designed a correction system to automatically measure and compensate the real-time circuit phase delay. This goal was achieved by collecting the amplitude of the automatic gain control (AGC) excitation signal and then automatically adjusting the phase reference value of the PLL. It was shown that this correction system is robust against the temperature change and that the compensated circuit phase delay was the true phase delay in the working process. Notably, we, for the first time, proposed a real-time circuit phase delay correction system that can accurately measure and compensate the circuit phase delay in real time to effectively eliminate the unwanted IQ coupling and reduce the effect of quadrature error on the ZRO, dramatically improving the BI and the ARW performance of the gyroscope.

The rest of this article is organized as follows. Section 2 describes the overview of the MEMS gyroscope dynamic. Section 3 gives the impact and theoretical analysis of circuit phase delay. The description of the real-time circuit phase delay correction system is given in Section 4. Finally, Section 5 presents the experimental results and analysis, and Section 6 concludes this paper.

## 2. MEMS Gyroscope Dynamic Overview

**Figure 1:**
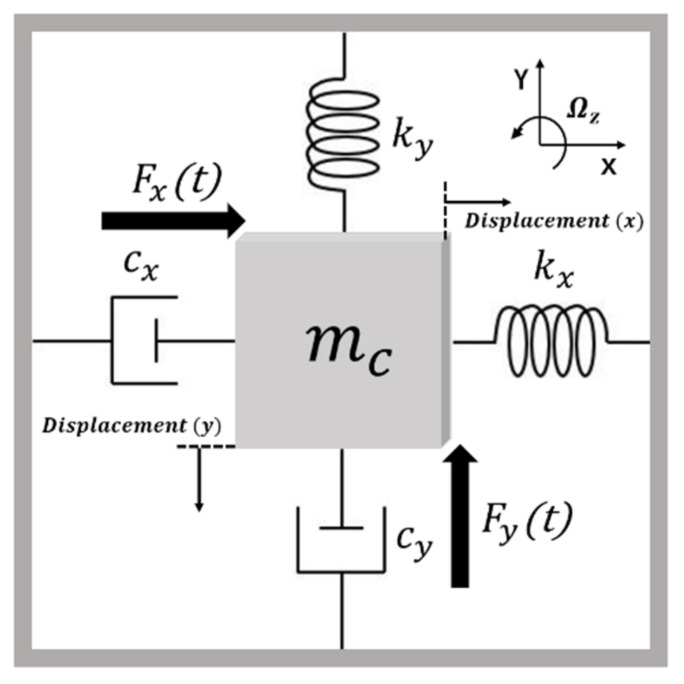
A simplified model of MEMS vibratory gyroscopes.

A simplified model of a z-axis MEMS vibratory gyroscope is shown in Figure 1, where the x-axis, the y-axis, and the z-axis are the drive, the sense, and the angular velocity input direction, respectively [23]. Additionally, $c_{x}$, $k_{x}$, $c_{y},\;\mathrm{and}\;k_{y}$ represent the damping coefficient and the spring stiffness coefficient on the *x*-direction and the *y*-direction. A MEMS vibratory gyroscope is composed of two orthogonal mechanical resonators, called the drive mode and the sense mode, which can be described as a mass-damper-spring second-order system.

Ideally, the simplified dynamical equations of the drive and sense model are revealed in Equations (1) and (2):

(1) $$m_{x}\ddot{x}+c_{x}\dot{x}+k_{x}x=F_{x}\left(t\right)$$

(2) $$m_{y}\ddot{y}+c_{y}\dot{y}+k_{y}y=F_{y}\left(t\right)-2m_{C}\Omega_{z}\dot{x}$$

where *x* and *y* are the vibration displacement in the drive mode and sense mode, $m_{x}$ and $m_{y}$ represent the effective mass in the *x*- and *y*-directions, respectively, which are equal to the Coriolis mass, $m_{C}$. $\Omega_{z}$ is the input angular velocity, $F_{x}\left(t\right)=A_{f}\cos\left(w_{d}t\right)$ is the drive force ($A_{f}$ is the excitation amplitude and $w_{d}$ is the drive force frequency), and $F_{y}\left(t\right)$ is the sense force (i.e., $F_{y}\left(t\right)=0$, when the sense mode is under open-loop). The expression of the drive resonant frequency $w_{x}$ and the drive quality factor $Q_{x}$ are:

(3) $$w_{x}=\sqrt{\frac{k_{x}}{m_{x}}}$$

(4) $$Q_{x}=\frac{m_{x}w_{x}}{c_{x}}$$

Substituting Equations (3) and (4) into (1) yields the transient-response $x\left(t\right)$ of the mass in the drive direction, which can be obtained by:

(5) $$x\left(t\right)=A_{x}\cos\left(w_{d}t+\varphi_{x}\right)$$

(6) $$|A_{x}|=\frac{A_{f}/m_{x}}{\sqrt{{\left(w_{x}^{2}-w_{d}^{2}\right)}^{2}+w_{x}^{2}w_{d}^{2}/Q_{x}^{2}}}$$

(7) $$\varphi_{x}=-\arctan\frac{w_{x}w_{d}}{Q_{x}\left(w_{x}^{2}-w_{d}^{2}\right)}$$

where $\left|A_{x}\right|$ is the amplitude of the vibration displacement and $\varphi_{x}$ is the drive phase delay. When the drive force frequency $w_{d}$ is accurately locked to the drive resonant frequency $w_{x}$ by the PLL, the vibration amplitude acquires the maximum value, and the phase delay $\varphi_{x}$ is just −90° [24]. It can be concluded from Equations (6) and (7) that with the difference between the drive force frequency $w_{d}$ and drive resonant frequency $w_{x}$ increasing, the vibration amplitude $\left|A_{x}\right|$ becomes smaller and the phase delay $\varphi_{x}$ becomes far away from −90° in the working process. Therefore, the phase delay $\varphi_{x}$ can be changed by adjusting the difference between $w_{d}$ and $w_{x}$.

## 3. Impact and Theoretical Analysis of Circuit Phase Delay

Considering the inevitable manufacturing imperfections, the drive shaft (*x*-direction) is not completely perpendicular to the sense shaft (*y*-direction), and the damping and stiffness coupling of the two modes cause the in-phase $\mathrm{force}\;F_{I}\left(t\right)$ and quadrature force $F_{q}\left(t\right)$. Equation (2) can be described as Equation (8):

(8) $$m_{y}\ddot{y}+c_{y}\dot{y}+k_{y}y+C_{xy}\dot{x}+k_{xy}x=F_{y}\left(t\right)-2m_{C}\Omega_{z}\dot{x}$$

where $C_{xy}$ is the coupling damping coefficient and $k_{xy}$ is the coupling spring stiffness. By taking Equation (5) into Equation (8), the sense dynamical equation can be given as:

(9) $$m_{y}\ddot{y}+c_{y}\dot{y}+k_{y}y=F_{y}\left(t\right)-2m_{C}\Omega_{z}A_{x}w_{d}\sin\left(w_{d}t+\varphi_{x}\right)\;-C_{xy}A_{x}w_{d}\sin\left(w_{d}t+\varphi_{x}\right)-k_{xy}A_{x}\cos\left(w_{d}t+\varphi_{x}\right)$$

The Coriolis effect is a special effect when a particle makes a composite motion with respect to inertial space. When the implicated motion of a particle is a rotational motion, the direction of the Coriolis acceleration $a_{c}$ is perpendicular to the direction of the input angular velocity $\Omega_{z}$ and the vibration velocity ${\vec{v}}_{c}$ ($a_{c}=2\left(\Omega_{z}\times {\vec{v}}_{c}\right)$ [25]. Hence, the in-phase force $F_{I}\left(t\right)=C_{xy}A_{x}w_{d}\sin\left(w_{d}t+\varphi_{x}\right)$ is proportional to the velocity, whereas the quadrature force $F_{q}\left(t\right)=k_{xy}A_{x}\cos\left(w_{d}t+\varphi_{x}\right)$ is proportional to the displacement of the drive mode [26].

**Figure 2:**
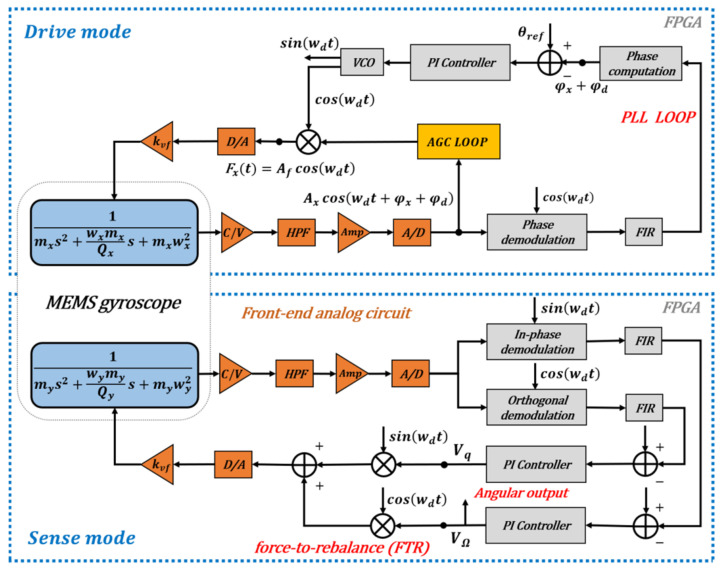
A schematic depiction for the circuit phase delay of MEMS vibration gyroscopes.

According to [1], the noise of the system itself does not affect the calculation of the circuit phase delay, so the noise component in the ZRO is ignored in the system phase analysis. Based on electrostatic drive and capacitance detection, the vibration gyroscope control system in this study was mainly composed of a front-end analog circuit, a signal processing and loop control based on FPGA, as shown in Figure 2. The front-end analog circuit is responsible for capacitor signal reading and conversion, signal amplification and filtering, and analog-to-digital (A/D) and digital-to-analog (D/A) conversion [27]. The loop control system mainly consisted of two parts: the drive mode control circuit composed of the PLL and AGC loops, which keep the gyroscope vibration amplitude and frequency stable, and an FTR closed-loop detection and quadrature error correction system, which is used to keep the sense mode relatively static and to collect the angular velocity signal.

When the signal goes through D/A, A/D, C/V conversion, amplification, filtering, and other modules of the control circuit, the circuit phase delay $\varphi_{d}$ is inevitable [28]. Without phase compensation (the reference value of the PLL is −90°), the existence of the PLL in the drive loop makes the difference between its input and output phase −90°. Therefore, the following phase relationship is satisfied:

(10) $$\varphi_{d}=\varphi_{DA}+\varphi_{cv}+\varphi_{HPF}+\varphi_{AD}+\varphi_{oth}$$

(11) $$\varphi_{d}+\varphi_{x}=-90^{{}^{\circ}}$$

where $\varphi_{DA}$, $\varphi_{cv},\;\varphi_{HPF}$, and $\varphi_{AD}$ are the phase delay of the DAC, C/V converter, high pass filter, and the ADC, respectively, and the $\varphi_{oth}$ is the sum of other factors that introduce phase delay, such as V/F conversion, interface, etc. As seen from Equation (11), the existence of the circuit phase delay $\varphi_{d}$ causes the drive phase delay $\varphi_{x}\neq -90^{{}^{\circ}}$. According to the previous analysis, shown in Equation (7), it can be concluded that the circuit phase delay $\varphi_{d}$ causes a frequency difference between the drive force frequency $w_{d}$ and the drive resonant frequency $w_{x}$, and the gyroscope is not working in the resonance state. Next, the impact of circuit phase delay $\varphi_{d}$ on the performance will be discussed in-depth, and a real-time circuit phase delay measurement and compensation system is proposed.

Considering the existence of circuit phase delay $\varphi_{d}$, the transient-response $x\left(t\right)$ of the driving direction changes, and Equation (5) can be rewritten as:

(12) $$x\left(t\right)=A_{x}\cos\left(w_{d}t+\varphi_{x}+\varphi_{DA}\right)$$

**Figure 3:**
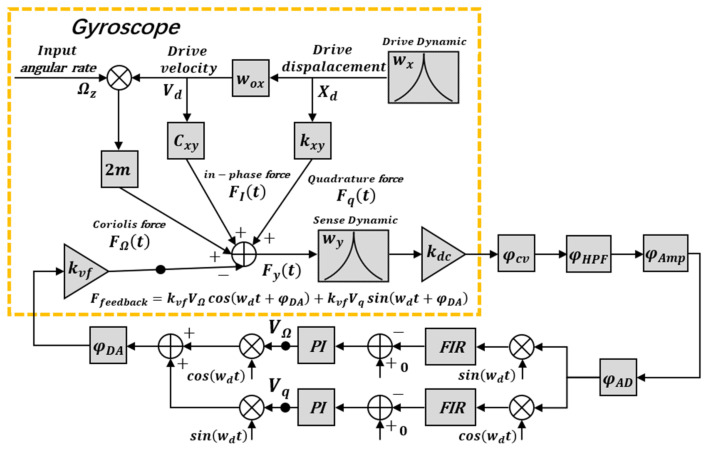
The phase system for the gyroscope sense model.

Under these circumstances, the sense dynamic Equation (9) is changed into:

(13) $$m_{y}\ddot{y}+c_{y}\dot{y}+k_{y}y=F_{y}\left(t\right)-2m_{C}\Omega_{z}A_{x}w_{d}\sin\left(w_{d}t+\varphi_{x}+\varphi_{DA}\right)\;-C_{xy}A_{x}w_{d}\sin\left(w_{d}t+\varphi_{x}+\varphi_{DA}\right)-k_{xy}A_{x}\cos\left(w_{d}t+\varphi_{x}+\varphi_{DA}\right)$$

where in-phase force $F_{I}\left(t\right)=C_{xy}A_{x}w_{d}\sin\left(w_{d}t+\varphi_{x}+\varphi_{DA}\right)$ and the quadrature force $F_{q}\left(t\right)=k_{xy}A_{x}\cos\left(w_{d}t+\varphi_{x}+\varphi_{DA}\right)$. As shown in Figure 3, the FTR closed-loop detection and quadrature error correction control mechanism cause the resultant force exerted on the sense proof mass to be nulled ($F_{y}\left(t\right)=0$):

(14) $$F_{\Omega }\left(t\right)+F_{I}\left(t\right)+F_{q}\left(t\right)=k_{vf}V_{\Omega }\cos\left(w_{d}t+\varphi_{DA}\right)+k_{vf}V_{q}\sin\left(w_{d}t+\varphi_{DA}\right)$$

$$2m_{C}\Omega_{z}A_{x}w_{d}\sin\left(w_{d}t+\varphi_{x}+\varphi_{DA}\right)+C_{xy}A_{x}w_{d}\sin\left(w_{d}t+\varphi_{x}+\varphi_{DA}\right)+k_{xy}A_{x}\cos\left(w_{d}t+\varphi_{x}+\varphi_{DA}\right)\;=k_{vf}V_{\Omega }\cos\left(w_{d}t+\varphi_{DA}\right)+k_{vf}V_{q}\sin\left(w_{d}t+\varphi_{DA}\right)$$

Hence, the in-phase channel feedback output $V_{\Omega }$ and the quadrature channel feedback output $V_{q}$ can be calculated:

(15) $$\left\{\begin{array}{l} V_{\Omega }=\frac{\left[\left(2m_{C}\Omega_{z}A_{x}w_{d}+C_{xy}A_{x}w_{d}\right)\sin\left(\varphi_{x}\right)+k_{xy}A_{x}\cos\left(\varphi_{x}\right)\right]}{k_{vf}}\\ V_{q}=\frac{\left[\left(2m_{C}\Omega_{z}A_{x}w_{d}+C_{xy}A_{x}w_{d}\right)\cos\left(\varphi_{x}\right)-k_{xy}A_{x}\sin\left(\varphi_{x}\right)\right]}{k_{vf}} \end{array}\right.$$

From Equation (15), the phase relationship between the two channels of the sense mode depends on the drive phase delay $\varphi_{x}$. When the drive force frequency $w_{d}$ is accurately locked to the drive resonant frequency $w_{x}$, the drive phase delay $\varphi_{x}$ is −90°, and the two channels are independent.

With the existence of the circuit phase delay $\varphi_{d}$, the drive phase delay $\varphi_{x}\neq -90^{{}^{\circ}}$ according to Equation (11), and the two channels are coupled to each other. Additionally, a portion of the quadrature error fluctuation will be introduced into the angular velocity detection output, which seriously affects the performance of the vibration gyroscope. In this time, the output of the gyroscope at zero angular velocity input ($\Omega_{z}=0$) is:

(16) $$V_{\Omega }=\frac{\left[C_{xy}A_{x}w_{d}\sin\left(\varphi_{x}\right)+k_{xy}A_{x}\cos\left(\varphi_{x}\right)\right]}{k_{vf}}$$

By substituting Equation (11) into Equation (16), the relationship between the in-phase output and the circuit phase delay $\varphi_{d}$ can be obtained as:

(17) $$V_{\Omega =0}=\frac{\left[-C_{xy}A_{x}w_{d}\cos\left(\varphi_{d}\right)-k_{xy}A_{x}\sin\left(\varphi_{d}\right)\right]}{k_{vf}}$$

It can be seen from the above derivation that the existence of the circuit phase delay $\varphi_{d}$ leads to the coupling of the in-phase and the quadrature channel (IQ coupling), which introduces an uncontrollable low-frequency noise, leading to a large drift in the ZRO. Besides, the IQ coupling will reduce the scale factor of the system and affect the response to angular velocity of the gyroscope, causing huge damage to the performance of the gyroscope, such as the BI and the ARW. In order to eliminate the influence of circuit phase delay on the performance, it is essential to compensate the circuit phase delay $\varphi_{d}$.

## 4. Real-Time Circuit Phase Delay Correction System

A real-time circuit phase delay correction system was proposed and was based on the amplitude-frequency and phase-frequency characteristics of the MEMS gyroscope and the phase relationship of the whole control loop. It can be concluded from Equations (6) and (7) that when the excitation amplitude $A_{f}$ is fixed, as the drive force frequency $w_{d}$ is closer to the drive resonant frequency $w_{x}$, the vibration amplitude $\left|A_{x}\right|$ is larger and the phase delay of the drive mode is closer to −90°. When the $w_{d}$ is equal to the $w_{x}$, the vibration amplitude $\left|A_{x}\right|$ acquires the maximum value, and the $\varphi_{x}$ is just −90°.

**Figure 4:**
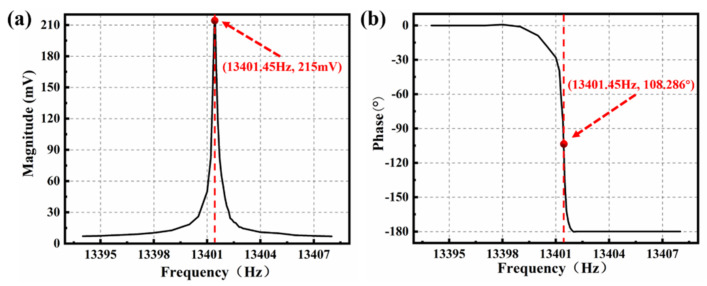
The (**a**) amplitude-frequency and (**b**) phase-frequency responses under the open-loop frequency sweep.

In order to verify these characteristics of the MEMS gyroscope, an open-loop frequency sweep test on the front-end analog circuit was carried out, as shown in [2]. The excitation signal with a fixed amplitude (100 mV) and a linearly increasing frequency (13,394–13,408 Hz; 0.05 Hz) was generated by FPGA programming, and the amplitude and phase response under each excitation were automatically collected. As shown in Figure 4a, when the frequency of excitation signal was 13,401.45 Hz, the amplitude response of the gyroscope reached its maximum value, and the phase delay between the excitation signal and the output signal was 108.286° in this case. The experimental results showed that it is feasible to study the phase delay of the gyroscope with amplitude-frequency and phase-frequency characteristics. This one-time frequency sweep method is easy to operate and implement, and the circuit phase delay $\varphi_{d}$ of the front-end analog circuit was estimated to be about 108.286° − 90° = 18.286° in this way. However, the measured phase delay was the off-line phase delay of the gyroscope, and it was unable to accurately measure the real phase delay in the working process, so the measured phase delay was not complete.

Based on the previous analysis, the drive force frequency $w_{d}$ and the drive resonant frequency $w_{x}$ can be gradually approached by compensating the circuit phase delay. When the phase delay $\varphi_{x}$ is 90°, the vibration amplitude $\left|A_{x}\right|$ reaches the maximum value, and the compensated phase delay is the circuit phase delay of the whole circuit. Because of an AGC loop, which was adopted to make the vibration amplitude $\left|A_{x}\right|$ stable, the amplitude-frequency Equation (6) can be reduced to Equation (18). Therefore, when $w_{d}$ and $w_{x}$ are gradually approached by compensating the circuit phase delay, the excitation amplitude $A_{f}$ becomes smaller, and the compensated phase delay is the real circuit phase delay $\varphi_{d}$ until it reaches the minimum value.

(18) $$A_{f}=|A_{x}|m_{x}\sqrt{{\left(w_{x}^{2}-w_{d}^{2}\right)}^{2}+w_{x}^{2}w_{d}^{2}/Q_{x}^{2}}$$

**Figure 5:**
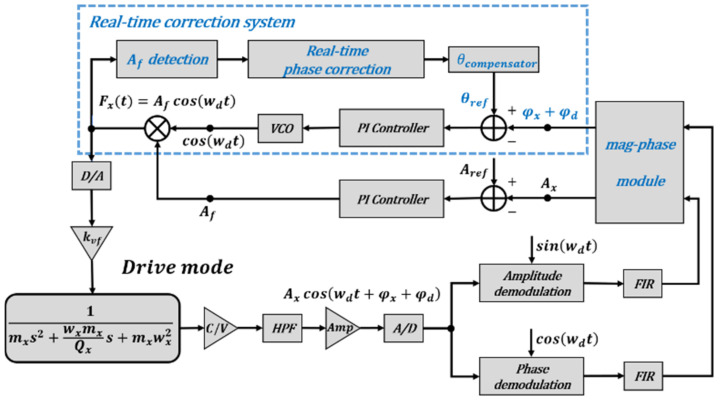
The diagram of the real-time circuit phase delay correction system.

In order to measure and compensate the circuit phase delay in the working process, a real-time circuit phase delay correction system was proposed based on the previous analysis. The diagram of this real-time correction system is shown in Figure 5, and the detailed process is described as follows:

(1) The gyroscope is in normal working condition: the PLL and AGC loops of the drive mode control circuit keep the gyroscope vibration amplitude and frequency stable, and an FTR closed-loop detection and quadrature error correction system is used to keep the sense mode relatively static and to collect the angular velocity signal.
(2) The gyroscope starts up normally.
(3) When the gyroscope is stable, the circuit phase delay correction system starts to work; the change of the excitation amplitude $A_{f}$ is automatically detected to adjust the phase compensation value ($\theta_{compensator}$) and the reference value of PLL ($\theta_{ref}$) is compensated gradually.
(4) Until the excitation amplitude $A_{f}$ reaches the minimum value, the correction system tends to be stable. At this time, the phase compensation value ($\theta_{compensator}$) under the negative feedback balance is the real-time circuit phase delay in the working process.
(5) The measurement and compensation of the circuit phase delay $\varphi_{x}$ are completed by the correction system, and the gyroscope is adjusted to the resonant working state.

A real-time circuit phase delay correction system was proposed, which can automatically measure and compensate for the circuit phase delay by detecting the excitation amplitude $A_{f}$ and adjusting the reference value of PLL ($\theta_{ref}$). When the circuit phase delay is compensated, the phase delay $\varphi_{x}$ is just −90° and the drive force frequency $w_{d}$ is exactly equal to the drive resonant frequency $w_{x}$.

## 5. Experimental Results and Analysis

**Figure 6:**
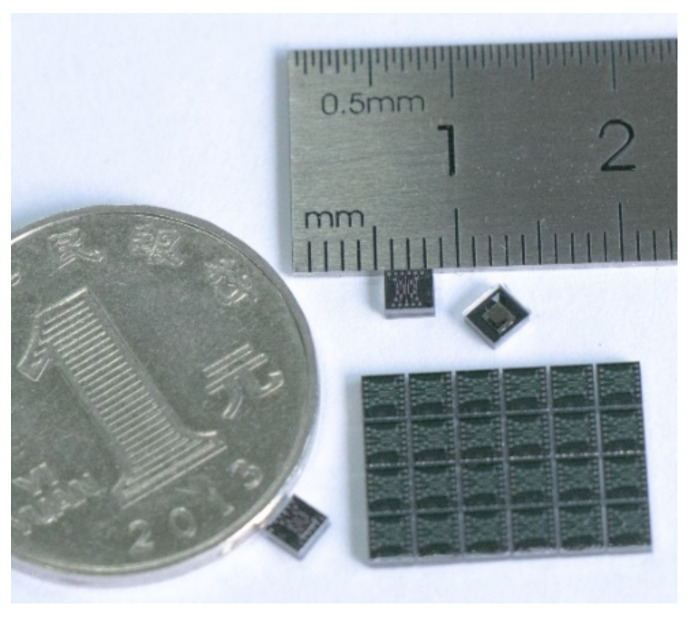
Photograph of the wafer-level-package capacitive vibration gyroscopes.

Self-developed shock resistant vibration gyroscopes were adopted in this study. A non-decoupled structure was designed to improve the impact resistance (about 100,000× *g*, which is crucial in tactical applications) of gyroscopes. To achieve large vibration amplitudes, the silicon wafers prefabricated with sensing structures were anodically bonded under a high vacuum pressure (about 0.8 mbar) at wafer level [29]. A photograph of the wafer-level-package (WLP) capacitive vibration gyroscopes is shown in Figure 6. The size of the gyroscopes was only 3 × 3 mm, and custom-made packages with metal lids were used to avoid the electrostatic charging caused by external sources [30]. The designed real-time circuit phase delay correction system was tested on these self-developed shock-resistant vibration gyroscopes. The circuit control system was mainly composed of an analog front-end circuit and a FPGA, and a USART was used for data acquisition at a 1 kHz sampling rate.

**Figure 7:**
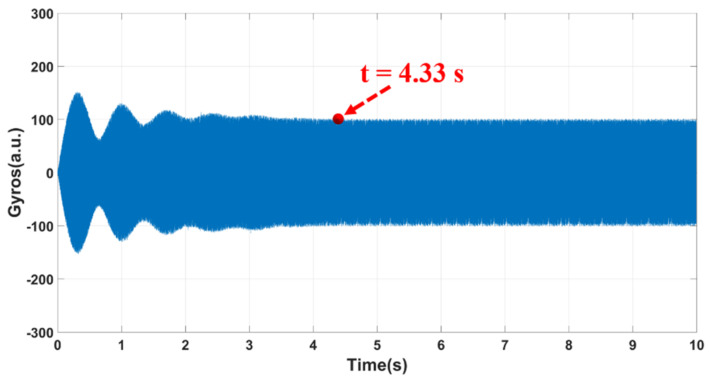
A diagram for the start-up process of the MEMS gyroscopes.

In order to effectively measure and compensate the circuit phase delay in the working process, the stability time of the MEMS gyroscope control loop was measured to avoid the interference between the real-time circuit phase delay correction system and the start-up process [31]. The test results are shown in Figure 7, where it can be seen that the turn-on time was about 4.33 s. Therefore, a 5 s delay was set in the correction system to ensure the normal operation of the MEMS gyroscope and to effectively measure and compensate the circuit phase delay in the working process.

**Figure 8:**
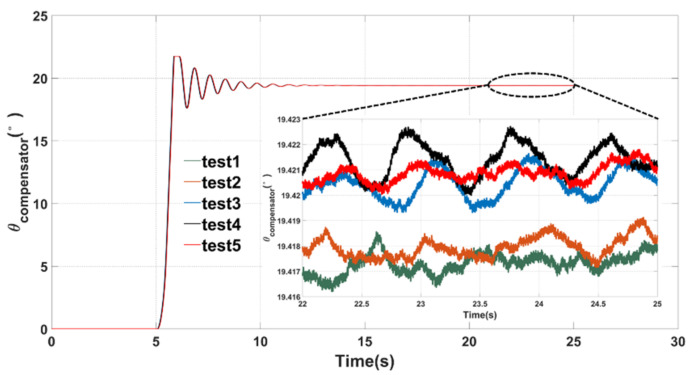
Test results of the real-time circuit phase delay correction system.

After the MEMS gyroscope works normally, the real-time circuit phase delay correction system starts to work, and the reference value of the PLL was automatically adjusted by collecting the change in the excitation amplitude $A_{f}$ until a negative feedback equilibrium was reached. In order to verify the effectiveness of the correction system, five repeated experiments were carried out. The test results are shown in Figure 8. This correction system had excellent repeatability and stability, which could effectively measure and compensate the real-time circuit phase delay within 10 s. The measured circuit phase delay was about 19.419 ± 0.004° in the working process.

**Figure 9:**
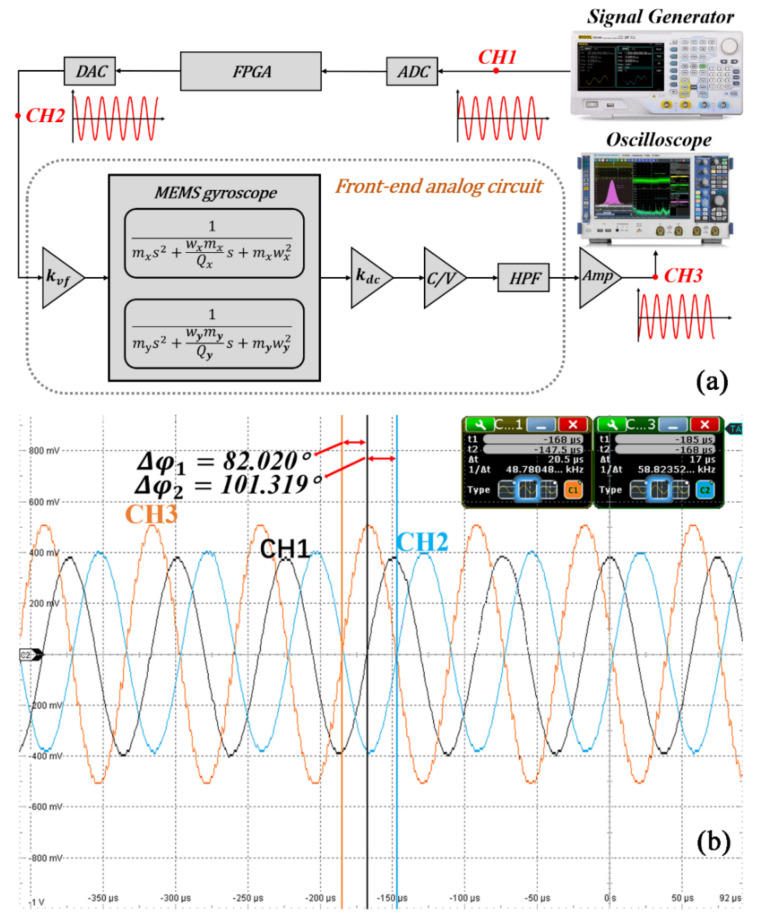
(**a**) The schematic diagram and (**b**) the test results of the one-time circuit phase delay measurement.

To investigate the feasibility of this real-time correction system, the circuit phase delay of the system was verified by the one-time measurement method, as shown in Figure 9a. The excitation frequency obtained from the open-loop frequency sweep test in Section 4, an excitation signal (CH1) with a frequency of 13,401.45 Hz and an amplitude of 400 mV, was generated by the signal generator (RIGOL DG4202). This signal was collected by the ADC, processed by the FPGA, and then passed through the DAC. After DC isolation, the MEMS gyroscope vibration and the vibration output signal went through CV conversion, filtering, and amplification. An oscilloscope (R&S ^®^RTO2000) was used to detect the phase change of the excitation signal. Figure 9b shows the test results of three channels (CH1, CH2, and CH3). The circuit phase delay of the signal processing modules (from the ADC to the DAC) was 90° − 82.020° = 7.98°, and the circuit phase delay of the analog front-end circuit was 101.319° − 90° = 11.319°. By calculation, the total circuit phase delay of the one-time measurement method was 7.98° + 11.319° = 19.299°. Comparing with the real-time circuit phase delay correction system, the deviation was only about 1%, which verified that this correction system can accurately and effectively measure the circuit phase delay.

**Figure 10:**
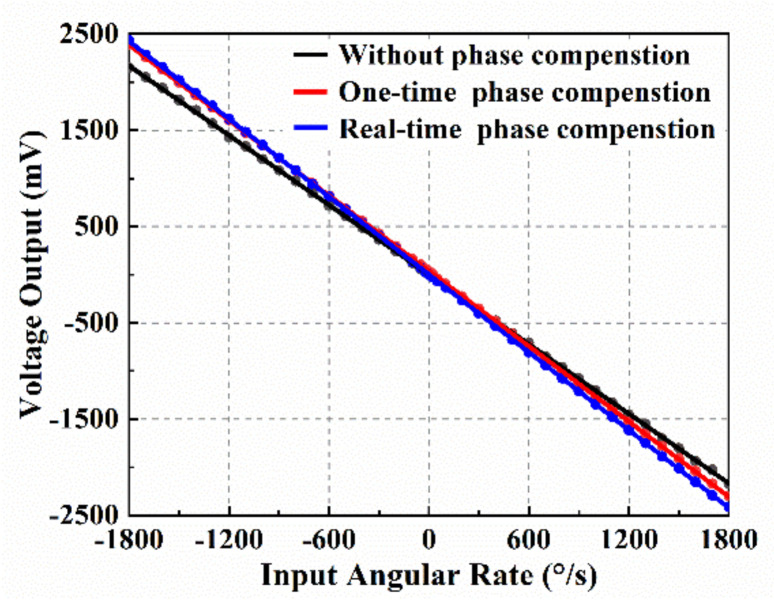
Test of the full scale and nonlinearity of the vibration gyroscope.

To measure the scale factor before and after phase delay compensation, the MEMS gyroscope and circuit system were placed on the angular velocity table (TBL-S1101-AT03). The continuous angular velocity rates, such as 0°/s, ±0.5°/s, ±1°/s, ±5°/s, ±10°/s, ±50°/s, ±100°/s, ±200°/s, and so on, were applied during the test, and each sampling point was collected for 1 min. The average value was taken as the corresponding angular rate output. As shown in Figure 10, the full-scale range of the gyroscopes was over 1800°/s, which has a high-rate resolution as low as 0.1°/s. Without phase compensation, the scale factor was 1.203 mV/(°/s) with a nonlinearity of 2600 ppm, and the scale factor was 1.303 mV/(°/s) with a nonlinearity of 660 ppm under the one-time phase delay compensation. The scale factor was 1.345 mV/(°/s) with a nonlinearity of 255 ppm under real-time phase delay compensation, which was about a 10.2 times improvement on the nonlinearity. Comparing with the no-phase delay compensation, the phase delay correction system can effectively improve the angular velocity response of gyroscopes by reducing the IQ coupling, and the nonlinearity of the scale factor was significantly improved.

**Figure 11:**
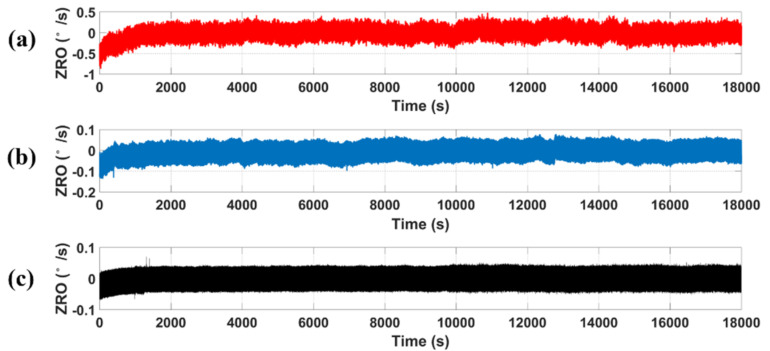
The ZRO acquired (**a**) without phase delay compensation and from (**b**) one-time and (**c**) real-time phase delay compensation at room temperature.

Figure 11 shows the measured ZRO at room temperature; the output angular rate was recorded at a 1 kHz sample rate for 5 h. Without phase delay compensation, the reference value $\theta_{ref}$ of the PLL was manually configured as −90°, and the $\theta_{ref}$ of the one-time phase delay compensation adopted the value −109.299° obtained from the previous one-time phase delay measurement. As shown in Figure 11a, the variation of ZRO was about 0.812°/s without phase delay compensation, and the ZRO of the one-time phase delay compensation was 0.14°/s, as shown in Figure 11b. The ZRO was improved about 8.55 times to 0.095°/s under the real-time phase delay compensation, as shown in Figure 11c. Experimental results showed that the real-time circuit phase delay correction system can effectively reduce the fluctuation of the output angular by eliminating the unwanted IQ coupling and reducing the effect of the quadrature error on the ZRO.

**Figure 12:**
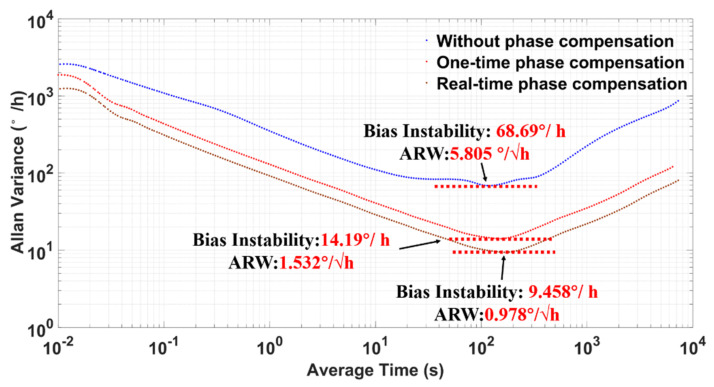
Allan variance plot at room temperature.

The Allan variance plot was obtained from the ZRO data collected previously (the ZRO data was measured at room temperature and recorded at a 1 kHz sample rate for 5 h) and is shown in Figure 12. It showed that the measured bias instability was 68.69°/h and that the ARW was 5.805°/√h without phase delay compensation. The bias instability was 14.19°/h and the ARW was 1.532°/√h under one-time phase delay compensation. Comparing with the no-phase delay compensation, the bias instability was increased by 7.26 times to 9.458°/h and the ARW was increased by 5.94 times to 0.978°/√h in the condition of the real-time phase delay compensation. The Allan variance results show that this phase delay correction system is effective in improving the BI and ARW performance of MEMS gyroscopes by eliminating the IQ coupling.

**Figure 13:**
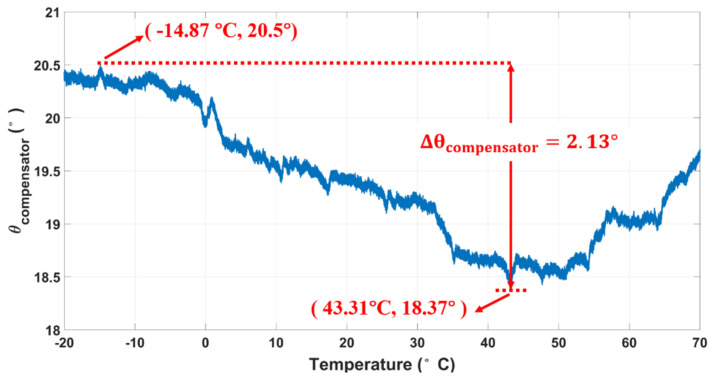
Measured circuit phase delay respect to temperature.

**Figure 14:**
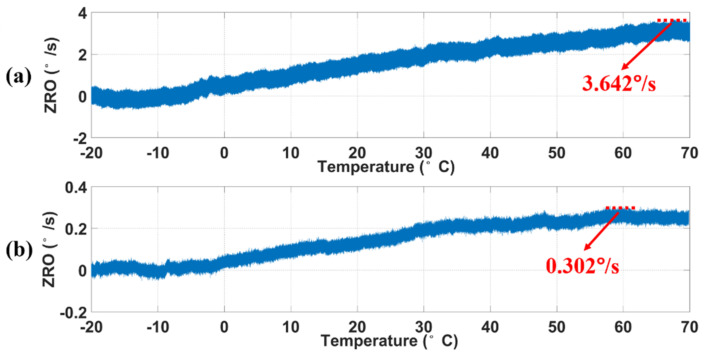
The measured ZRO (**a**) without and (**b**) with phase delay compensation with respect to temperature.

The performance of MEMS gyroscopes is directly affected by the change in ambient temperature, and the phase delay of the amplifier, resistor, capacitor, and processor modules in the circuit is affected by temperature. To verify the compensation effect of the real-time circuit phase delay correction system in this case, the MEMS gyroscopes and circuit system were placed in (HK-GDW-80), and the temperature measurement range was from −20 to 70 °C. As shown in Figure 13, the circuit phase delay decreased first and then increased with the change in temperature, and the maximum phase variation was about 2.13°. The variation curves of the ZRO without phase compensation are shown in Figure 14a. The ZRO gradually rose with the increase in temperature and reached 3.642°/s at 70 °C. However, the trend of the ZRO gradually slowed down with real-time phase compensation, and the maximum variation was only 0.302°/s, as shown in Figure 14b. The temperature coefficient (TCO) of the ZRO reduced from 0.04 to 0.0034°/s/°C and increased about 12.1 times. Experimental results showed that the real-time circuit phase delay correction system can effectively track the variation in circuit phase delay with the change in environment temperature, which realizes the precise measurement and compensation of the circuit phase delay. This correction system was robust against the temperature change and can effectively reduce the influence of temperature change on the performance of MEMS gyroscopes. 

Table 1 shows the summary of the measured performance with and without phase compensation. The real-time circuit phase delay correction system can effectively eliminate the unwanted IQ coupling and reduce the effect of the quadrature error on the ZRO through accurately measuring and compensating the circuit phase delay in real time. Ultimately, the ZRO, the scale factor, the BI, the ARW, and the TCO of the ZRO were significantly improved. Besides, all the acronyms in this paper are listed and shown in Table A1 in the Appendix A.

## 6. Conclusions

In summary, a real-time circuit phase delay correction system was proposed to automatically measure and compensate the circuit phase delay for MEMS vibratory gyroscopes. The effect of circuit phase delay on IQ coupling and the ZRO was analyzed under an FTR closed-loop detection and quadrature error correction system. By accurately measuring and compensating the real-time circuit phase delay, the correction system can effectively eliminate the unwanted IQ coupling and can greatly improve the performance of MEMS gyroscopes. This correction system achieved a decreased ZRO down to 0.095°/s, a small ARW of 0.978°/√h, and a low BI of 9.458°/h together with a scale factor nonlinearity of 255 ppm at room temperature. It was shown that the correction system is robust against the temperature change and that the thermal drifts of the ZRO were reduced to 0.0034°/s/°C. Our work presents a novel method to measure and compensate the circuit phase delay in real time, which may promote the evolution of high-performance MEMS gyroscopes for potential applications.

## Figures and Tables

**Table 1:** The performance of MEMS gyroscopes with and without phase delay compensation.

	Without Phase Compensation	One-Time Phase Compensation	Real-Time Phase Compensation	Improve
Zero rate output (°/s)	0.812	0.14	0.095	8.55
Scale factor (mV/(°/s))	1.203	1.303	1.345	1.12
Nonlinearity (ppm/°C)	2600	660	255	10.2
Bias instability (°/h)	68.69	14.19	9.458	7.26
Angle random walk (°/√h)	5.805	1.532	0.978	5.94
TCO of ZRO (°/s/°C)	0.04	---	0.0034	12.1

## Data Availability

Not applicable.

## Appendix A

**Table A1:** The list of acronyms and designations in the article.

Acronym	Designation
MEMS	micro-electromechanical system
IQ coupling	in-phase and quadrature coupling
ZRO	zero rate output
FTR	force-to-rebalance
PLL	phase-locked loop
BI	bias instability
ARW	angle random walk
AGC	automatic gain control
ADC	analog-to-digital
DAC	digital-to-analog
WLP	Wafer-Level-Package
TCO	temperature coefficient
